# Transcriptome profiling revealed candidate genes, pathways and transcription factors related to nitrogen utilization and excessive nitrogen stress in perennial ryegrass

**DOI:** 10.1038/s41598-022-07329-7

**Published:** 2022-03-01

**Authors:** Yinruizhi Li, Mengdi Wang, Ke Teng, Di Dong, Zhuocheng Liu, Tiejun Zhang, Liebao Han

**Affiliations:** 1 Turfgrass Research Institute, College of Grassland Science, Beijing Forestry University, Beijing, China; 2 Beijing Research and Development Center for Grass and Environment, Beijing Academy of Agriculture and Forestry Sciences, Beijing, China

**Keywords:** Plant sciences, Plant stress responses

## Abstract

Ryegrass (*Lolium perenne* L.), a high-quality forage grass, is a good nutrient source for herbivorous livestock. However, improving nitrogen use efficiency and avoiding nitrate toxicity caused by excessive nitrogen are continual challenges in ryegrass production. The molecular mechanism underlying the response of ryegrass to nitrogen, especially excessive nitrogen, remains unclear. In this study, the transcriptomic changes under different nitrogen levels were investigated in perennial ryegrass by high-throughput next-generation RNA sequencing. Phenotypic characterization showed that treatment with half of the standard N concentration (N0.5) led to a better growth state than the other three treatments. The treatments with the standard N concentration (N1) and treatments with ten times higher than the standard N concentration (N10) contained excessive nitrogen, which placed stress on plant growth. Analysis of differentially expressed genes indicated that 345 and 104 genes are involved in the regulation of nitrogen utilization and excessive nitrogen stress, respectively. KEGG enrichment analysis suggested that “photosynthesis-antenna proteins” may respond positively to appropriate nitrogen conditions, whereas “steroid biosynthesis”, “carotenoid biosynthesis” and “C5-branched dibasic acid metabolism” were identified as the top significantly enriched pathways in response to excessive nitrogen. Additionally, 21 transcription factors (TFs) related to nitrogen utilization were classified into 10 families, especially the AP2-EREBP and MYB TF families. Four TFs related to excessive nitrogen stress were identified, including LOBs, NACs, AP2-EREBPs and HBs. The expression patterns of these selected genes were also analyzed. These results provide new insight into the regulatory mechanism of ryegrass in response to nitrogen utilization and excessive nitrogen stress.

## Introduction

Nitrogen is the main nutrient element needed for plant growth and plays a very important role in promoting crop yield. Increased application of nitrogen fertilizer is one of the measures necessary to achieve high yield. However, excessive application of nitrogen fertilizer not only reduces plant nitrogen use efficiency but also causes a series of problems, such as reduced crop planting efficiency, resource waste and environmental pollution^1^. It is very important to determine the appropriate nitrogen fertilizer application rate for the cost-effective, environmentally friendly and sustainable development of agriculture. With the development of molecular biology, many genes related to nitrogen utilization have been identified and cloned, helping in further elucidation of the molecular mechanism of nitrogen uptake and utilization in crops and laying a foundation for improving nitrogen use efficiency in crops by genetic engineering.

The processes that occur when nitrogen enters plants include absorption, transport and distribution. Two transporter gene families have been implicated in nitrogen uptake in plant roots: the nitrate transporter 1/small peptide transporter family NPF (NRT1/PTR) and NO_3_^−^ transporter family (NRT2). The NRT1 and NRT2 families in *Arabidopsis thaliana* include 53 and 7 genes, respectively. NRT1 is a subset of the low-affinity absorption and transport system (LATS) for NO_3_, and NRT2 is a component of the high-affinity absorption and transport system (HATS) for NO_3_^–^^2^. When the external NO_3_^−^ concentration was lower than 1 mmol·L^−1^, the HATS played a major role in regulating the inward flow of NO_3_^−^, and when the external NO_3_^−^ concentration was greater than 0.5 mmol·L^−1^, the LATS started to play a role^3^. The mechanism underlying the absorption of NH_4_^+^ by plant roots was similar to that for NO_3_^−^ and included a HATS and an LATS. The HATS of NH_4_^+^ depends on the AMT gene family, which is divided into the AMT1 and AMT2 subfamilies, present in root epidermal cells and the membrane of the cortex^4^. In addition, four NO_3_^−^ transporter families have been found to be involved in the transport, distribution and utilization of NO_3_^−^ in plants: the NPF (NRT1/pTR) family, NRT2 family, chloride channel family (CLC), and s-type anion channels and their homologues (SLAC/SLAH)^5^. Although significant progress has been made in understanding the physiology and molecular biology of nitrogen uptake, transport, and distribution and their regulation in plants, currently, research focuses mainly on certain common plants whose performance is strongly affected by the environment, whereas little is known about the molecular mechanism of the response to nitrogen in other species with relatively narrow application ranges. For ryegrass, previous studies showed that under salt stress, the NRT gene in annual ryegrass seedlings did not respond to moderately low N application^6^. The expression of *LpNRT1.1* under high-nitrogen conditions was generally higher than that under low-nitrogen conditions, suggesting that NRT1.1 may play an important role in the LATS^7^.

Perennial ryegrass (*Lolium perenne* L.) is a high-quality forage grass that can be a good nutrient source for herbivorous livestock and promote the development of animal husbandry^8^. However, as a grass family member, perennial ryegrass itself does not have the ability to fix nitrogen, and the nitrogen required for its growth and development comes mainly from the soil via absorption by root systems. The nitrogen available in the soil is often insufficient for meeting the needs of forage grass. Therefore, supplementing soil nitrogen with fertilizer is one of the most effective measures for producing high-quality and high-yield forage. It should be noted that increasing artificial nitrogen fertilizer application does not necessarily result in more available nitrogen for absorption by plants. When nitrogen application exceeds a certain level, nitrogen use efficiency decreases, and nitrate is enriched in plants, leading to poisoning of grazing livestock feeding on plants with high nitrate levels^9^. Therefore, it is of great importance to study the endogenous molecular mechanism underlying nitrogen utilization and the response to excessive nitrogen stress in perennial ryegrass, which can provide a theoretical foundation for guiding production practices.

In recent years, studies on nitrogen in ryegrass have focused on its effect on the yield and quality of forage grass, livestock and agricultural byproducts. A mini-sward study conducted in Valdivia found that the lower the frequency of nitrogen application was, the higher the total crude protein content of ryegrass, but the crude protein component was not affected by the application frequency^10^. Another study showed that applying high levels of nitrogen to a tetraploid or diploid perennial ryegrass meadow mixed with white clover effectively increased milk production per hectare per cow^11^. The effects of nitrogen on several plant transcriptomes have been characterized, such as those on the rice^12^, potato^13^, *Arabidopsis*^14^, wheat^15^, sorghum^16^, poplar^17^, nankingense^5^, and tea^18^ transcriptomes. However, some of these studies focused on the carbon and nitrogen balance at the transcriptional level, and the others focused on the change in transcriptional levels under nitrogen deficiency. However, the molecular mechanism underlying the response of ryegrass to nitrogen has rarely been reported.

In this study, we used high-throughput next-generation DNA sequencing (NGS) technology to profile the transcriptome of perennial ryegrass under different nitrogen levels to screen candidate genes related to nitrogen utilization and stress and to further understand the molecular mechanisms related to nitrogen in ryegrass. On the basis of previous studies, our study explored changes in the transcription levels of ryegrass under excessive nitrogen levels, which compensated for the molecular mechanism of the plant response to nitrogen to some extent.

## Results

### Phenotypic characterization of perennial ryegrass under different concentrations of nitrogen

**Figure 1:**
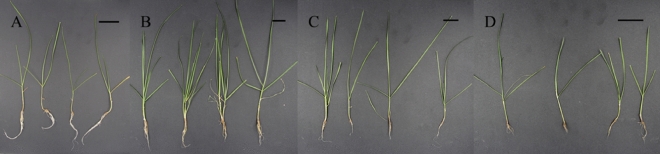
Growth state of perennial ryegrass under different treatments. (**A**–**D**) represent treatments N0, N0.5, N1, and N10, respectively. The black scale bar in each figure indicates 3 cm.

**Figure 2:**
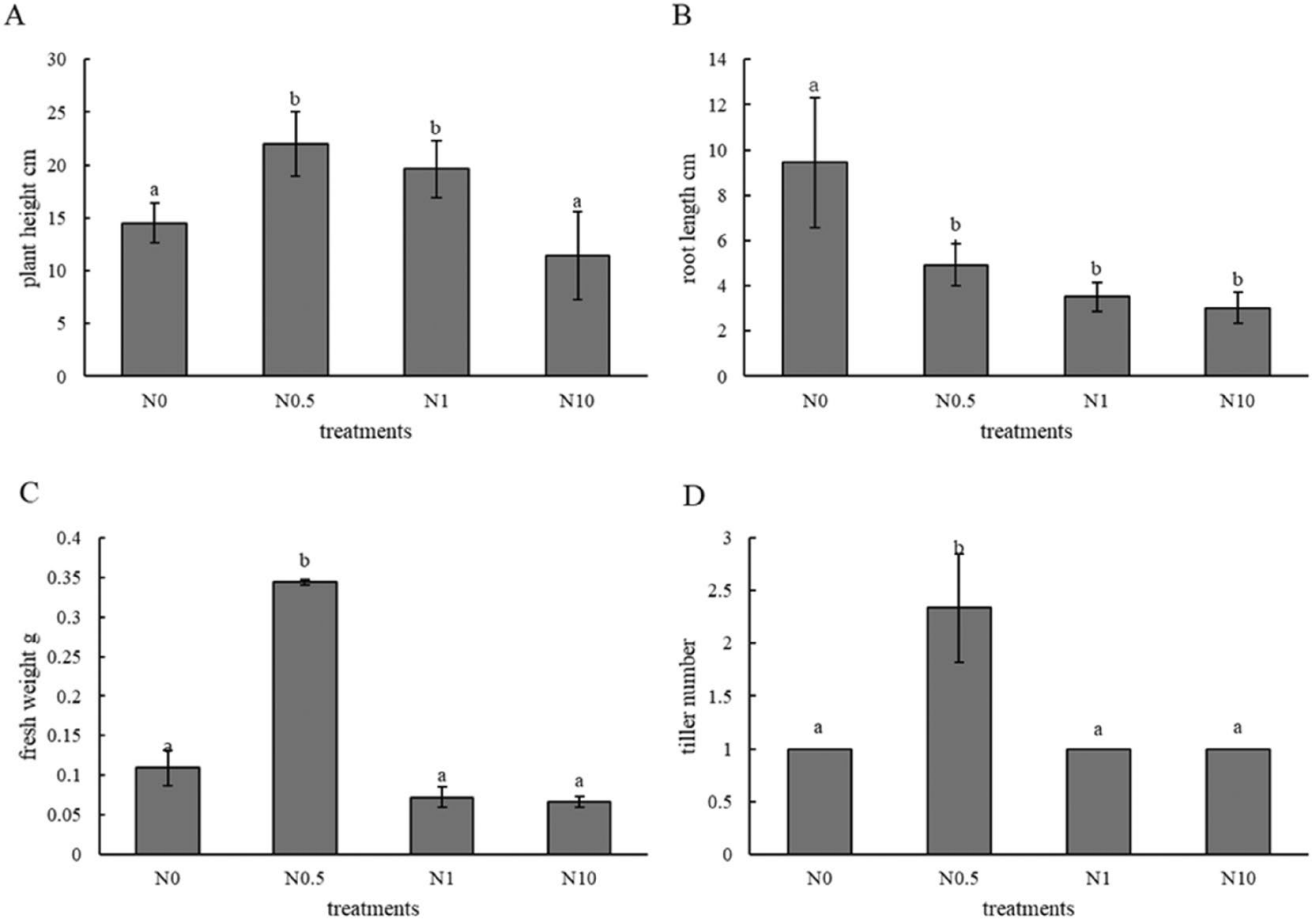
Physiological characteristics of the plants. (**A**–**D**) represent plant height, root length, fresh weight and tiller number of different treatments in turn.

**Figure 3:**
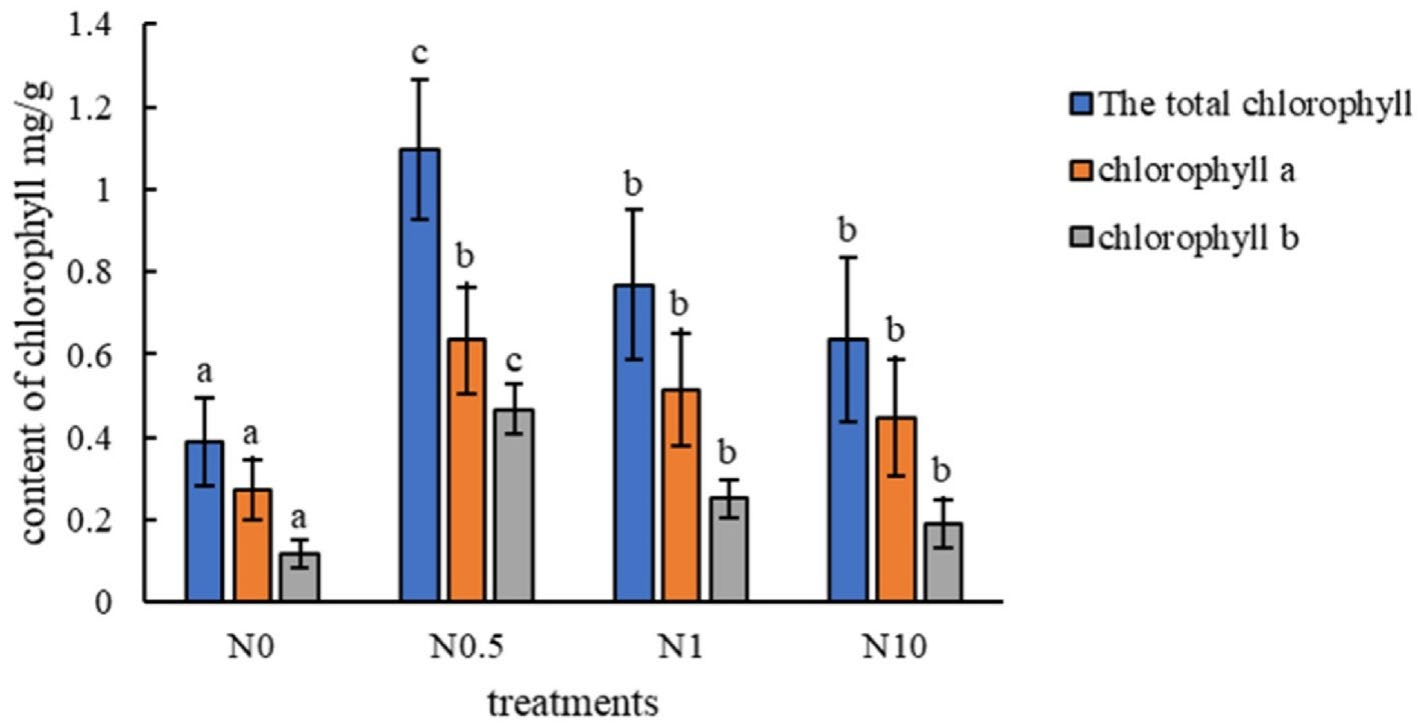
Chlorophyll content. The blue columns indicate the total chlorophyll content. The yellow and grey columns indicate the chlorophyll *a* and *b* levels, respectively. The letters a and b in all the figures represent significant differences, and error bars indicate differences between biological replications.

To describe the growth state of perennial ryegrass under different treatments (Fig. 1), the plant height, root length, fresh weight, tiller number and chlorophyll content of all samples were determined. The results showed that the plant heights in treatments N0.5 and N1 were significantly higher than those in the other two treatments (Fig. 2A). The fresh weight and tiller number in the N0.5 treatment were significantly higher than those in the other treatments (Fig. 2C,D). However, the root length in treatment N0 as a control was the highest and was significantly higher than that in the other treatments (Fig. 2B). The results of chlorophyll content determination indicated that there was a significant increase in chlorophyll content under treatment N0.5 compared to that in the other three groups; moreover, the chlorophyll content was shown to first increase and then decrease in all four treatments (Fig. 3). Based on the above, it could be inferred that ryegrass exhibited better growth under treatment N0.5 than under the other three treatments. Treatments N1 and N10 contained excessive nitrogen, which stressed plant growth.

### Sequencing and transcriptome assembly

To comprehensively identify the transcripts of perennial ryegrass in response to nitrogen, 12 cDNA libraries were constructed from the four treatment groups (N0, N0.5, N1, N10) corresponding to the conditions of no nitrogen, a moderate amount of nitrogen, moderately excessive nitrogen and highly excessive nitrogen, each of which had three biological replications. In total, 80.58 GB of clean data was obtained, and the amount for each sample varied from 6.02 to 7.20 GB (Table S1). All clean data were subjected to genomic localization analysis (http://185.45.23.197:5080/ryegrassgenome) by HISAT. In each sample, the proportion of mapped clean reads varied between 61.84% and 68.12%, while the fraction of multiple mapped reads was between 0.66 and 0.86% (Table S2). Through transcriptome assembly, these transcripts were classified into 101,376 unigenes, including 2185 novel genes (Table S3). The raw data were loaded into the SRA database of the NCBI (BioProject ID: PRJNA660099) (Table S4).

### Analysis of differentially expressed genes (DEGs)

**Figure 4:**
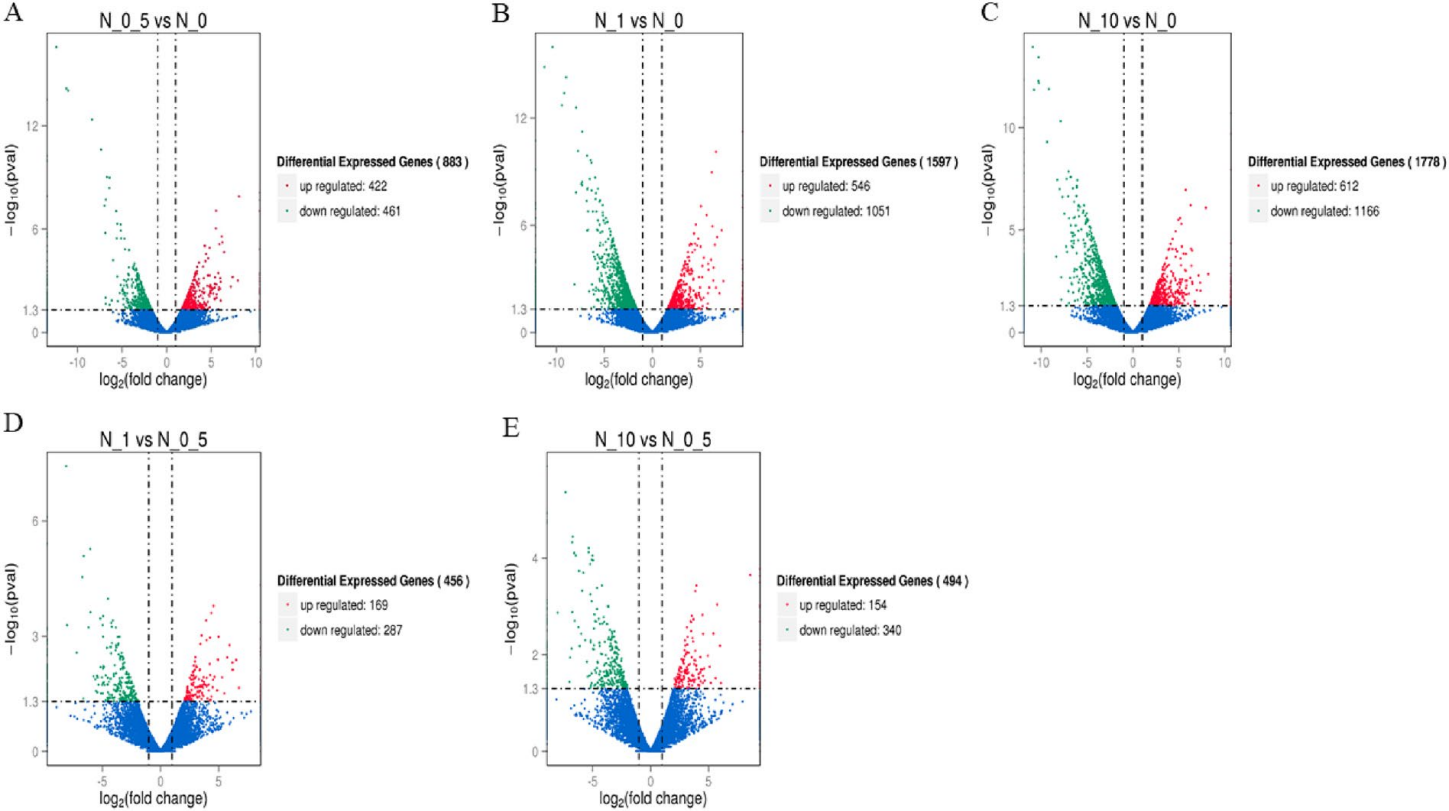
Volcano plot of the differentially expressed genes (DEGs). (**A**–**C**) represent DEGs among three treatment groups (N0.5, N1 and N10), with treatment N0 as a control. (**D**,**E**) represent DEGs among two treatments (N1 and N10), with treatment N0.5 as a control. Genes with significant differential expression are represented by red dots (upregulated) and green dots (downregulated), while genes without significant differential expression are represented by blue dots. The abscissa represents the multiple changes in gene expression in different samples. The ordinate represents the statistical significance of the difference in gene expression.

To understand the response of ryegrass genes to nitrogen, DEGs in three treatments (N0.5, N1 and N10) were screened, with treatment N0 as a control. Under these conditions, 883, 1597, and 1778 genes were identified in treatments N0.5, N1 and N10, respectively. In treatment N0.5, 422 genes were upregulated and 461 genes were downregulated. Accordingly, in treatment N1, 546 genes were upregulated and 1051 genes were downregulated. A total of 642 genes were upregulated and 1166 genes were downregulated in treatment N10 (Table S5A1–2, B1–2, C1–2, Fig. 4A–C).

**Figure 5:**
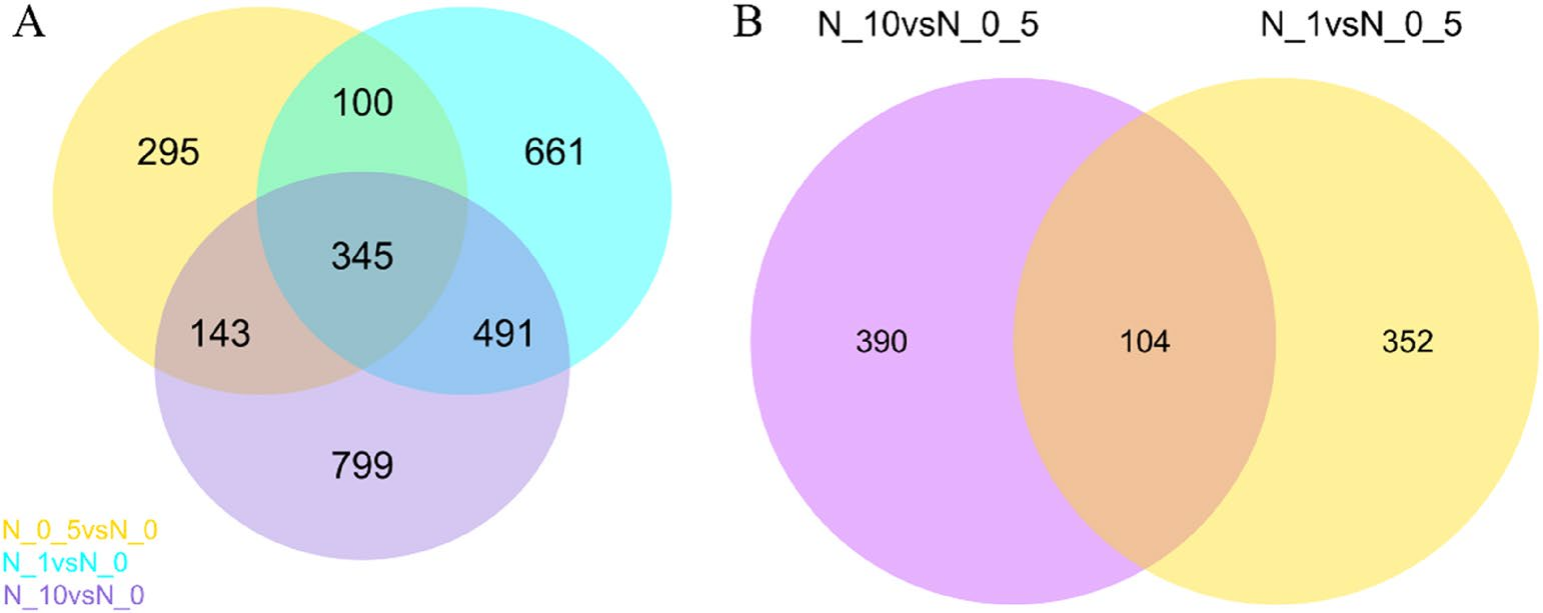
Venn diagram of DEGs. Panel (**A**) represents the DEGs in the three treatment groups compared to treatment N0 as a control. Panel (**B**) represents the DEGs in treatments N1 and N10 compared to treatment N0.5 as a control. The sum of the numbers in each large circle represents the total number of DEGs in the comparison combination, and the overlapping circles represent the number of DEGs in common between the combinations.

The differential expression of these genes was caused by the addition of nitrogen. To identify more genes related specifically to nitrogen utilization, the three groups of DEGs were visualized as a Venn diagram (Fig. 5A). The results indicated that 345 genes were directly involved in the regulation of nitrogen utilization, among which 322 genes are known genes, while the rest are novel genes (Table S6).

In terms of plant growth status, the N0.5 treatment was more suitable for ryegrass growth than the other three treatments, and treatments N1 and N10 produced excessive nitrogen stress in the plants. Based on this, we further attempted to screen genes that specifically respond to excessive nitrogen stress. DEGs in treatments N1 and N10 were identified using treatment N0.5 as a control (Table S5D1–2, E1–2, Fig. 4D,E). There were 456 (169 upregulated, 287 downregulated) and 494 (154 upregulated, 340 downregulated) DEGs in treatments N1 and N10, respectively. Furthermore, 82 known genes and 22 novel genes were identified among the common genes in treatments N1 and N10 compared to treatment N0.5 (Table S7, Fig. 5B). These common genes may play an important role in the response of perennial ryegrass to excessive nitrogen stress.

### Clustering of unigenes related to nitrogen utilization and excessive nitrogen stress

**Figure 6:**
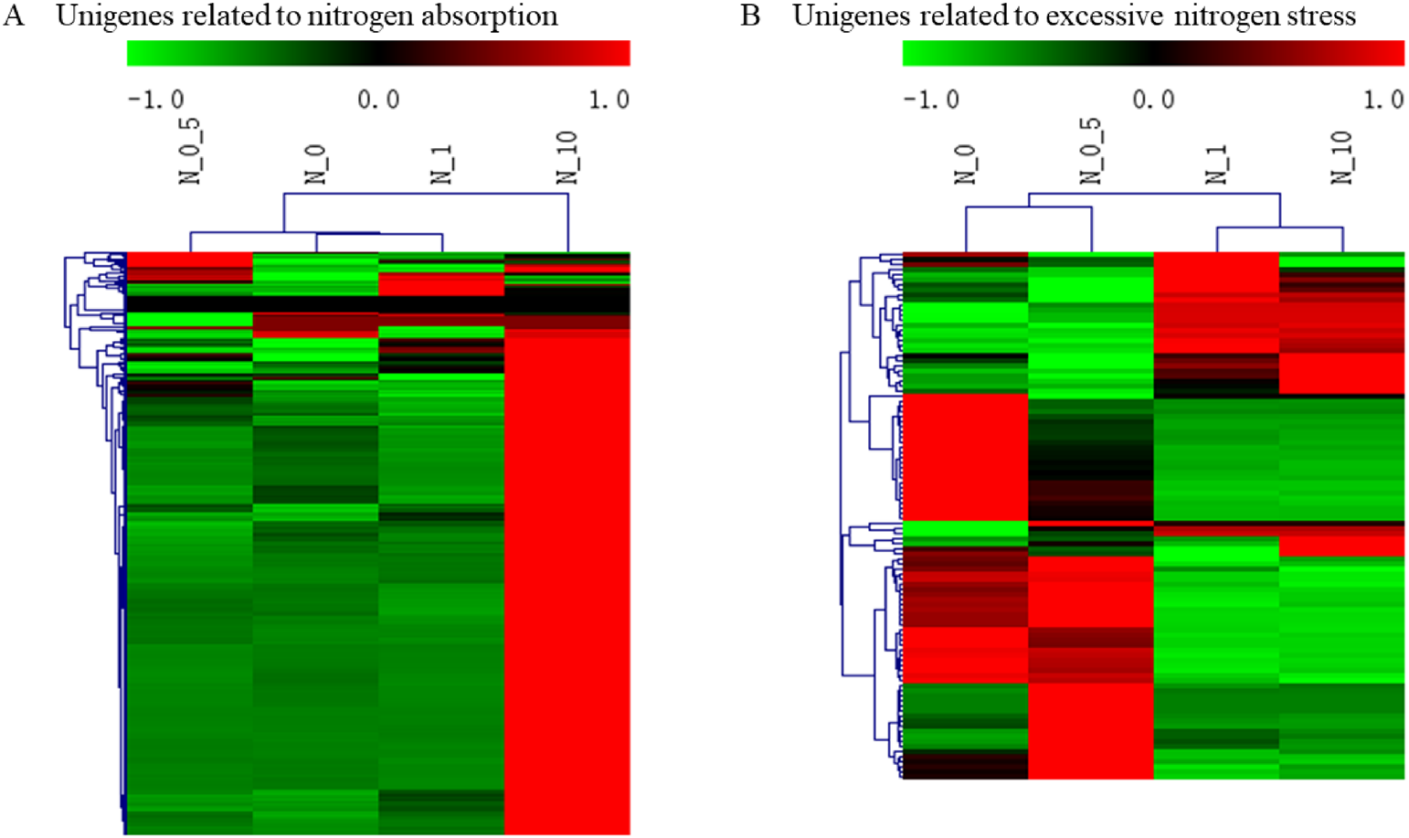
Heat map clustering analysis of unigenes related to nitrogen utilization and excessive nitrogen stress. (**A**) and (**B**) show the clustering of unigenes related to nitrogen utilization and excessive nitrogen stress, respectively. High-expression genes are shown in red, and low-expression genes are shown in green.

To gain insights into the expression patterns of the screened unigenes related to nitrogen utilization and excessive nitrogen stress, their expression levels in the different nitrogen treatment groups were clustered separately (Fig. 6). Clustering analysis of unigenes related to nitrogen utilization revealed that the expression of the genes in treatment N0.5 was most similar to that in treatment N1, followed by that in treatment N0 and that in treatment N10 (Fig. 6A). Considering the nitrogen content in the different treatments, presumably, these genes exhibited analogous expression in response to a moderate amount of nitrogen and moderately excessive nitrogen but distinct expression in response to highly excessive nitrogen. It is worth mentioning that most of the genes related to nitrogen utilization had relatively high expression in treatment N10, whereas they had relatively low expression in the other three groups. This result suggested that most of these genes exhibited similar expression patterns.

On the other hand, the genes related to excessive nitrogen stress exhibited similar expression levels between treatment N0.5 and treatment N0, and a similar result was observed for treatment N1 and treatment N10 (Fig. 6B). It could be inferred that the genes related to excessive nitrogen stress were expressed homoplastically under the condition of no nitrogen and a moderate amount of nitrogen, considering the different nitrogen levels among treatments. Similarly, these genes exhibited analogous expression in response to excessive nitrogen ranging from a moderate concentration to a high concentration.

### KEGG enrichment analyses of DEGs

**Figure 7:**
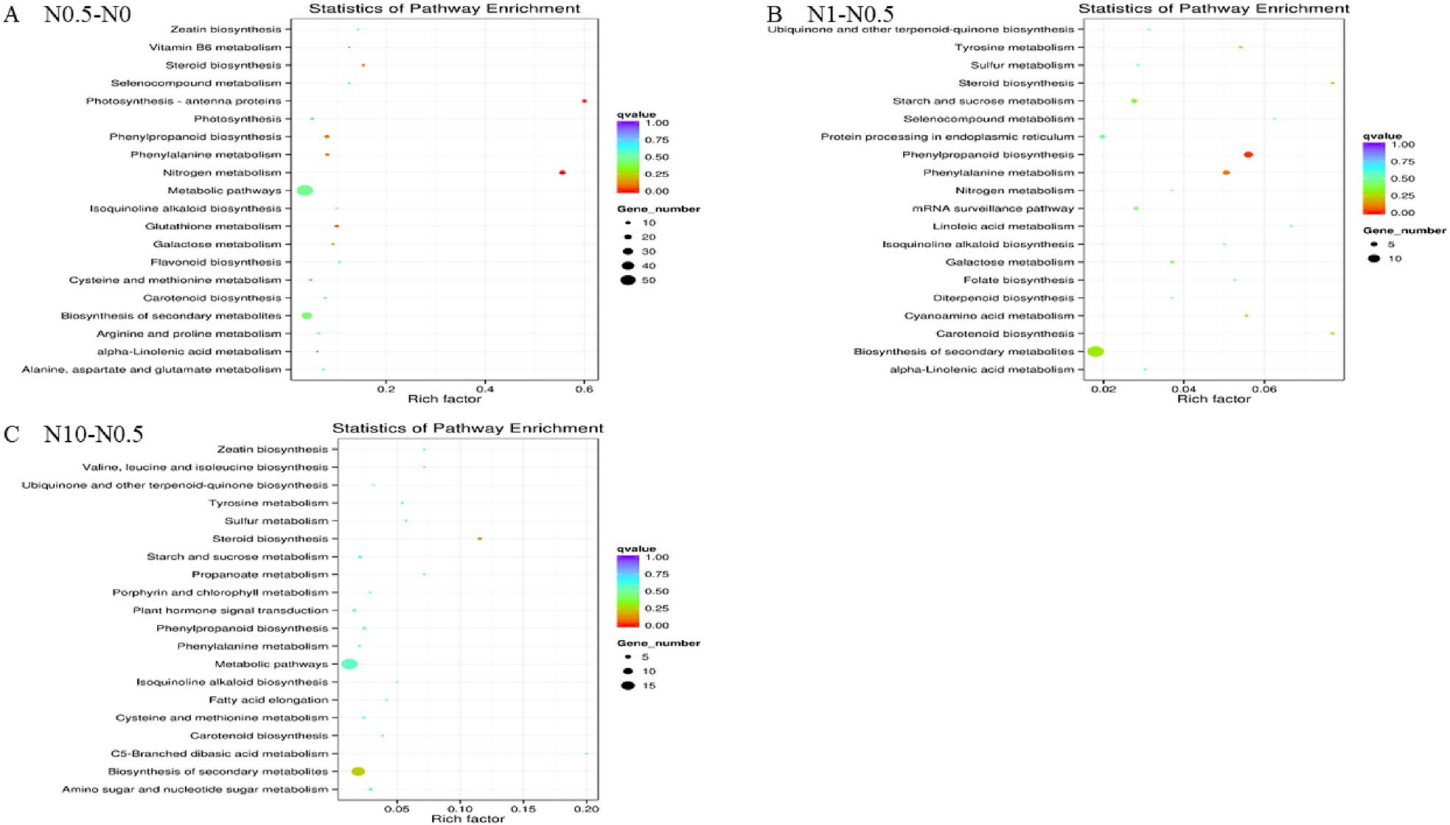
KEGG enrichment distribution of DEGs. (**A**) shows the enrichment of DEGs in the pathway in the N0.5 treatment versus N0. (**B**,**C**) show the N1 treatment versus N0.5, and the N10 treatment versus N0.5, respectively. The vertical axis represents the pathway name, the horizontal axis represents the rich factor, the size of the dots represents the number of DEGs in the pathway, and the colour of the dots corresponds to different q-value ranges.

**Figure 8:**
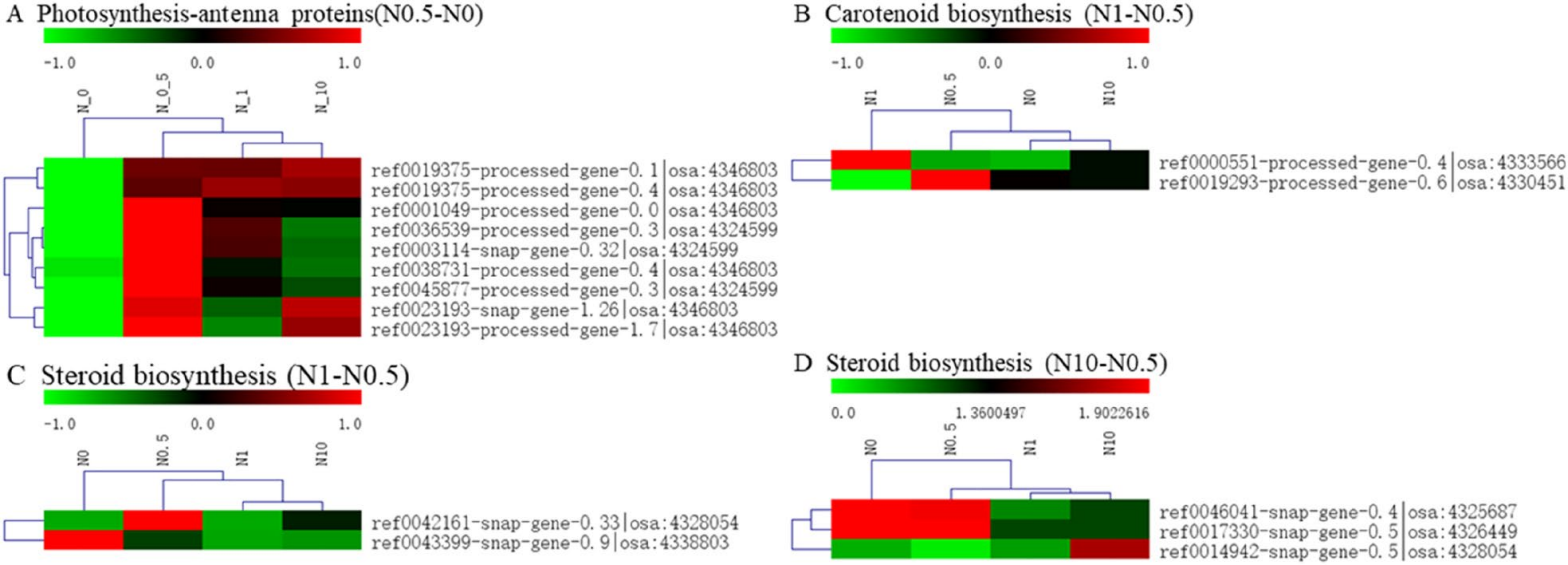
Heat map clustering analysis of DEGs enriched in three pathways. (**A**) represents the clustering of DEGs enriched in the photosynthesis-antenna protein pathway in treatment N0.5 compared to treatment N0. (**B**, **C**) represent the clustering of DEGs enriched in the steroid biosynthesis pathway and carotenoid biosynthesis pathway in treatment N1 compared to treatment N0.5. (**D**) represents the clustering of DEGs enriched in the steroid biosynthesis pathway in treatment N10 compared to treatment N0.5. High-expression genes are shown in red, and low-expression genes are shown in green.

DEGs were separately enriched in 60, 37 and 38 pathways (Tables S8–S10, respectively). Twenty pathway items with the most significant enrichment are displayed in Fig. 8. DEGs in treatments N0.5 and N1 (Fig. 6A,B) displayed a certain degree of enrichment in the “nitrogen metabolism” pathway. The results for treatment N0.5 compared to N0 showed that the top enriched pathway of the DEGs was “photosynthesis-antenna proteins”, not “nitrogen metabolism” (Fig. 7A). This result suggested that “photosynthesis-antenna proteins” may also respond positively to nitrogen under appropriate nitrogen conditions. Similarly, “steroid biosynthesis” and “carotenoid biosynthesis” were identified as the top two significantly enriched pathways in the KEGG enrichment analysis of DEGs between treatments N1 and N0.5 (Fig. 7B). In addition, there were two relatively highly enriched pathways (“phenylpropanoid biosynthesis”, “phenylalanine metabolism”) with lower q-values (Fig. 7B). This indicated that genes in these specific pathways play an important role in the response mechanism of plants to a certain amount of nitrogen stress. Regarding the pathways enriched in treatment N10 compared to N0.5, the “C5-branched dibasic acid metabolism” (only Novel00490|osa:4328147 enriched) and “steroid biosynthesis” pathways showed the most significant enrichment (Fig. 7C). Thus, “steroid biosynthesis” may perform some unknown and irreplaceable functions under excessive nitrogen stress.

### Photosynthesis-antenna protein, steroid biosynthesis and carotenoid biosynthesis pathways

In the KEGG enrichment analysis of the DEGs, four specific pathways with significantly enriched DEGs were found in the three DEG groups associated different nitrogen levels. To further explore the response of these pathways to different nitrogen conditions, the enriched DEGs were mapped to those filtered pathways, and their expression was analysed by clustering (Fig. 8). There were 9, 2, 2, and 3 DEGs with significant enrichment classified into the photosynthesis-antenna protein, steroid biosynthesis and carotenoid biosynthesis pathways. These DEGs involved in the three specific pathways displayed relatively broad expression patterns. Notably, the different DEGs in treatments N1 and N10 compared to treatment N0.5 were significantly and simultaneously participated in the steroid biosynthesis pathway. This could indicate that the diverse parts of the steroid biosynthesis pathway function in the perennial ryegrass response to excessive nitrogen concentrations ranging from moderate to high.

### Nitrogen metabolism pathway

By KEGG enrichment analysis, we found 15 DEGs that responded to the standard amount of nitrogen in ryegrass and shared homology with the previously identified genes in the nitrogen metabolism pathway (Table S8). Twelve of them were successfully matched in the set of genes (Table S6), and we screened them as genes related to nitrogen utilization. This was due to the different methods used for statistical analysis of the data. In the same way, 1 and 0 DEGs related to a moderate amount of nitrogen stress and high concentration of nitrogen stress, respectively, exhibited homology to the previously identified genes in the nitrogen metabolism pathway (Tables S9 and S10). The DEG related to a moderate amount of nitrogen stress was not included in the gene collection that we screened as genes related to excessive nitrogen stress (Table S7). Considering that we screened 345 and 104 DEGs as candidate genes related to nitrogen utilization and excessive nitrogen stress, respectively (Tables S6 and S7), it was reasonable to assume that the remaining 437 DEGs were related to the nitrogen response specifically in ryegrass, excluding the 12 DEGs enriched in the nitrogen metabolism pathway.

**Figure 9:**
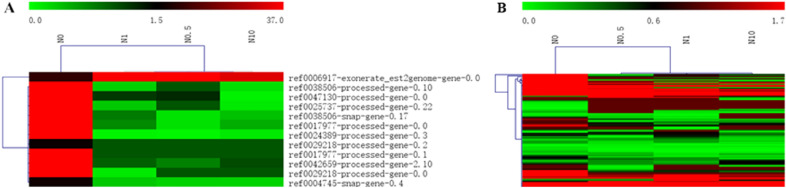
Heat map clustering analysis of DEGs involved in the nitrogen metabolism pathway and the NRT family. (**A**) represents the clustering of DEGs enriched in the nitrogen metabolism pathway. (**B**) represents the clustering of DEGs classified into the nitrogen transporter gene family. High-expression genes are shown in red, and low-expression genes are shown in green.

The expression analysis of these 12 DEGs showed that they exhibited a similar expression pattern, being upregulated in the N0 treatment and downregulated in the other treatments, except ref0006917-exonerate_est2genome-gene-0.0, which showed the reverse trend (Fig. 9A). This indicated that ref0006917-exonerate_est2genome-gene-0.0 might play a positive role in nitrogen utilization, while the remaining genes perform some important functions in the absence of nitrogen.

### NRT family

A total of 209 members of the transcriptome that were homologous to the NRTs in *Arabidopsis* were extracted, and 104 of them were differentially expressed in each treatment (Table S11). The expression patterns of these DEGs varied, but they were more closely expressed in the N0.5, N1, and N10 treatments than in the N0 treatment (Fig. 9B). This suggested that these NRTs might perform different functions in the absence of nitrogen compared to the presence of nitrogen.

### Identification of TFs

**Figure 10:**
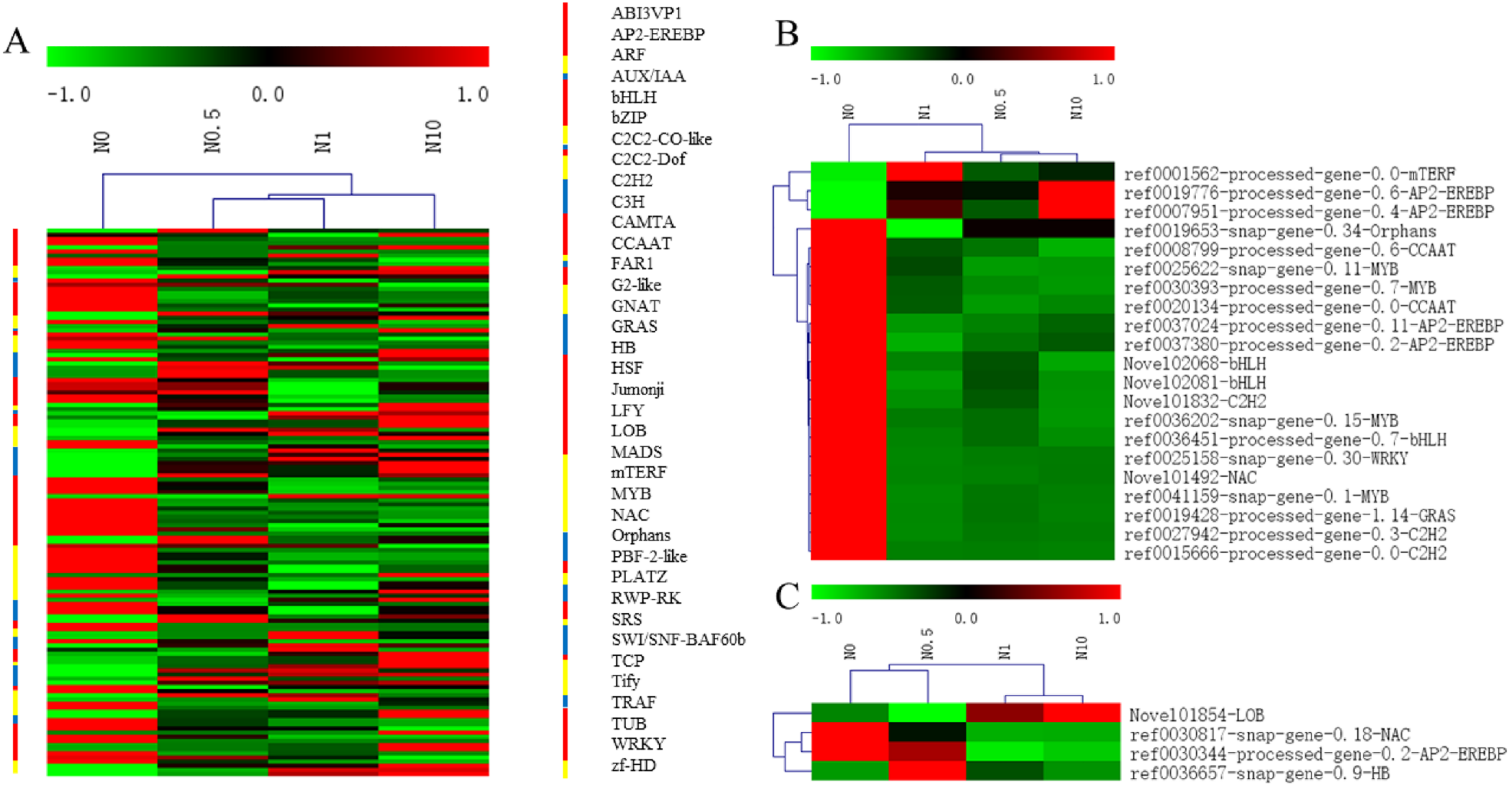
Heat map clustering analysis of TFs related to nitrogen, nitrogen utilization and excessive nitrogen stress. (**A**) represents the clustering of TFs responses to nitrogen in perennial ryegrass. (**B**) represents the clustering of TFs related to nitrogen utilization. (**C**) represents the clustering of TFs related to excessive nitrogen stress. The coloured columns indicate the different TF families, and their lengths indicate the number of TFs. High-expression genes are shown in red, and low-expression genes are shown in green.

There is accumulating evidence that transcription factors (TFs) play key roles in various regulatory mechanisms. Therefore, TFs among the DEGs were identified to further study the molecular regulatory network of perennial ryegrass in response to nitrogen (Fig. 10A). In total, 185 TFs, among which there were 14 novel genes, were identified among all the DEGs. These genes belonged to 37 TF families, including the top three TF families (AP2-EREBP, MYB, NAC), with greater than ten genes each.

**Figure 11:**
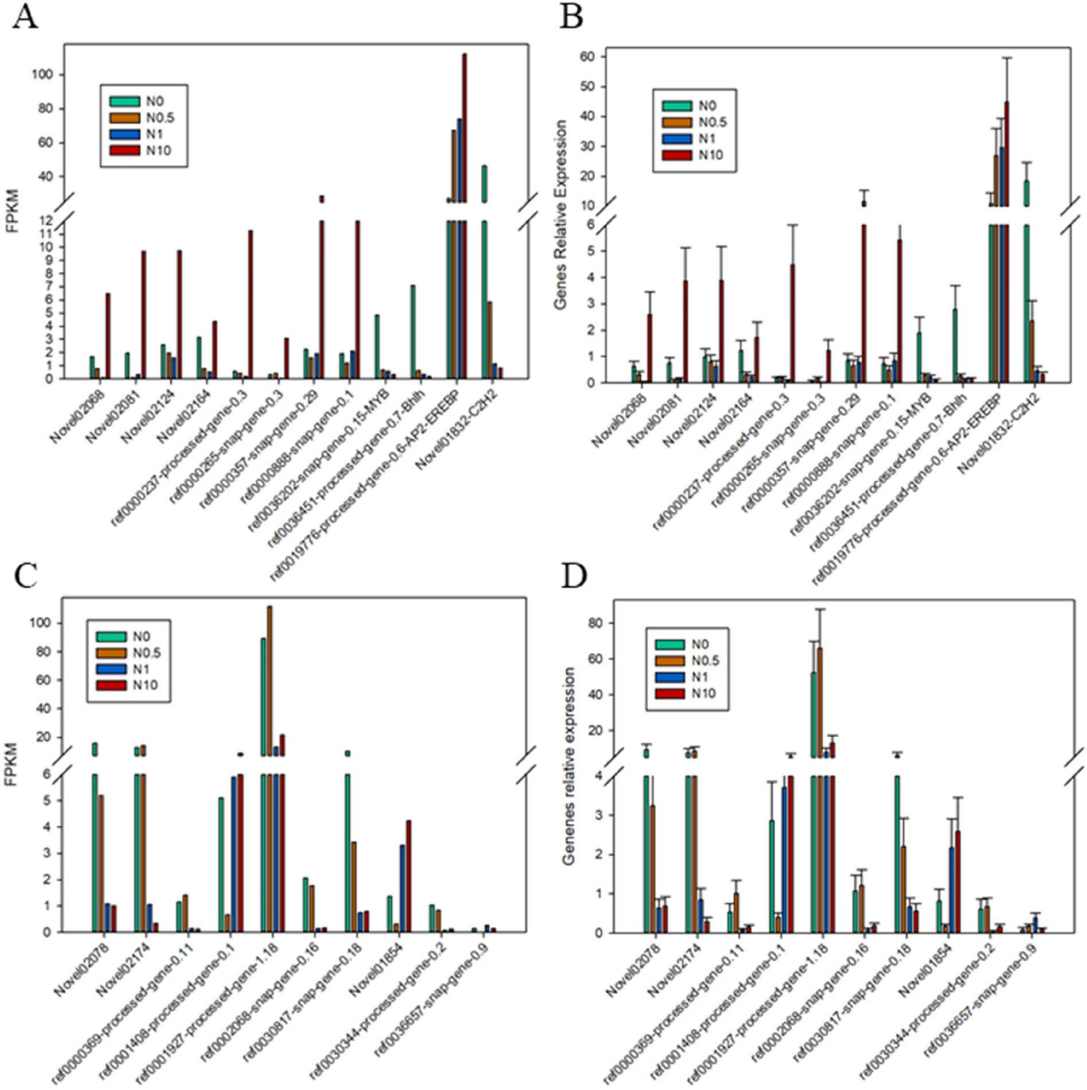
Expression of genes selected for validation. (**A** and **C**) represent FPKMs of genes related to nitrogen utilization and excessive nitrogen stress, as determined by RNA-Seq. (**B** and **D**) represent the relative expression of genes related to nitrogen utilization and excessive nitrogen stress, as determined by qRT–PCR.

The expression patterns of these TFs changed over a relatively large range. Based on this, TFs related to nitrogen utilization and excessive nitrogen stress were characterized (Figs. 10B, 11C). In total, 21 TFs related to nitrogen utilization were classified into 10 TF families, among which the AP2-EREBP and MYB TF families occupied the largest proportion (Fig. 10B). These TFs exhibited similar expression in treatments N0.5, N1 and N10, and most of them showed similar expression patterns in all treatments. Accordingly, four TFs belonging to 4 families related to excessive nitrogen stress were identified, including LOBs, NACs, AP2-EREBPs and HBs (Fig. 10C). The expression of these TFs was relatively similar in treatments N1 and N10, and two TFs in the HB family had similar expression patterns. Notably, ref0030344-processed-gene-0.2 from the AP2-EREBP family concurrently exhibited a positive response to nitrogen absorption and excessive nitrogen stress.

### Expression validation by qRT–PCR

To verify the accuracy of the expression data from RNA-Seq, 22 unigenes with significantly different expression levels were randomly selected for qRT–PCR analysis. These unigenes, including 12 DEGs related to nitrogen utilization and 10 related to excessive nitrogen stress, included eight TFs and 14 functional genes. The expression patterns of almost all of these genes in the qRT–PCR analysis results were consistent with the transcriptome profiling (Fig. 11). Thus, the qRT–PCR results were consistent with those from RNA-Seq, reflecting the high reliability and accuracy of the transcriptome data.

## Discussion

The response of plants to nitrogen, a key nutrient element for plant growth and development, involves a complex regulatory network with various kinds of functional genes and TFs. Elucidating these intricate molecular regulatory mechanisms would make great contributions to science-based application of nitrogen in production practices. In this study, to provide insight into the mechanisms of the perennial ryegrass response to nitrogen, we focused on the genes induced by nitrogen utilization and excessive nitrogen stress. The transcripts of perennial ryegrass cultivated at different concentrations of nitrogen were analysed by RNA-Seq, mainly for the identification of DEGs related to nitrogen utilization and excessive nitrogen stress.

Under moderate amounts of nitrogen, the plant heights, fresh weights and tiller numbers were higher than those under other conditions. On the other hand, it has been suggested, to some degree, that 0.5 times the standard Hoagland nutrient solution in treatment N0.5 provides the plants with a relatively appropriate amount of nitrogen. Analogously, there is evidence that increasing nitrogen supply leads to increasing plant biomass in nitrogen-deficient soil environments. For example, the biomass of sugarcane leaves increased significantly with increasing nitrogen fertilizer amounts^19^. Compared with the low-nitrogen condition, paper birch grew faster under the high-nitrogen condition in all the treatment groups; the total biomass increased, and the root/shoot ratio decreased. However, relatively long roots were observed in the no nitrogen treatment group than in the other groups. Existing research suggests that plants accumulate more carbon-assimilating substances into the root system to promote root growth and development to obtain more restricted resources, resulting in an increase in the root/shoot ratio of plants when soil nutrients are scarce^19^. Regarding the chlorophyll levels in perennial ryegrass under different concentrations of nitrogen, the level under the condition of 0.5 times the standard Hoagland nutrient solution was highest, and there was an overall trend of an increase and then a decrease in the four treatments. Previous research has shown that under nitrogen deficiency, the chlorophyll content in plant leaves decreases, and the F686/F740 fluorescence ratio increases^20^. Excessive nitrogen transfer of nutrients in maize resulted in premature leaf senescence and decreased photosynthetic capacity^21^. Above all, this suggests that the four nitrogen concentrations designed to represent the conditions of no nitrogen, a moderate amount of nitrogen, moderately excessive nitrogen and highly excessive nitrogen in the different treatments were relatively reasonable.

A total of 345 screened genes related to nitrogen utilization generally displayed analogous expression patterns, with a rapid increase in expression in cells treated with high concentrations of excessive nitrogen. The logical speculation that the expression of these genes would increase with increasing nitrogen supply and that their overexpression may cause a certain degree of disorder of nitrogen metabolism in plants under high concentrations of excessive nitrogen stress requires further verification. In addition, the expression patterns of 104 DEGs related to excessive nitrogen stress can be broadly divided into five categories. This indicates that these genes may regulate the plant response to excessive nitrogen stress in five corresponding ways.

Specific pathways that respond positively to different concentrations of nitrogen, including the photosynthesis-antenna protein pathway, were observed under moderate amounts of nitrogen in the KEGG enrichment analysis. Fifteen DEGs identified under moderate amounts of nitrogen application were assigned to 2 categories, including CAB1R (osa:4346803) and CAB2R (osa:4324599), encoding two types of light-harvesting complex II chlorophyll a/b binding proteins based on the KEGG database. A previous study showed that OsCSP41b has an impact on leaf colour in rice^22^. It can be inferred that the expression of these genes is induced by moderate amounts of nitrogen, and some analogous photosynthetic characteristics of perennial ryegrass were observed in this study. These DEGs may act as an important link between nitrogen and plant photosynthetic regulation mechanisms.

DEGs in the carotenoid biosynthesis pathway, including NCED|osa:4333566 and CCD|osa:4330451, showed enrichment under a certain amount of excessive nitrogen. OsNCED3 participates in seed dormancy, plant growth, abiotic stress tolerance and leaf senescence by regulating the biosynthesis of abscisic acid (ABA) in rice^23^. CitCCD4 converts β-cryptoxanthin and zeaxanthin to β-citraurin, leading to the flavedo of citrus fruit^24^. The novel functions of NCED and CCD identified in this study under excessive nitrogen stress remain to be explored.

Diverse parts of the steroid biosynthesis pathway have been found to function in the perennial ryegrass response to excessive nitrogen ranging from a moderate concentration to a high concentration (CYP15G1|osa:4328054 and SMT1|osa:4338803 under a moderate concentration and delta(7)-sterol-C5(6)-desaturase|osa:4325687, probable 3-beta-hydroxysteroid-Delta(8) |osa:4326449, and CYP|osa:4328054 under a high concentration). Based on previous studies, application of ammonium sulfate promotes the high expression of FPPS, SMT1, SMT2, SMO1, SMO2, ODM and other pathway genes, accompanied by an increase in the content of sterol required for anilide biosynthesis in *Withania somnifera* (L.) Dunal^25^. CYP51s catalyse the 14α-demethylation of sterol in all eukaryotes^26^. We can infer that these genes enriched in the steroid biosynthesis pathway are indeed regulated by exogenous nitrogen, and 14α-demethylation of sterol may be one of the key links between this pathway and excessive nitrogen stress.

A total of 16 DEGs were enriched in the nitrogen metabolism pathway and shared homology with the previously identified genes. Specifically, ref0006917-exonerate_est2genome-gene-0.0 exhibited a unique expression pattern and was upregulated as the nitrogen concentration increased. This gene encodes a kind of carbonic anhydrase belonging to the carbonic anhydrase (CA) family. Its main function is to accelerate the interconversion of CO_2_ to HCO_3_^27^. This suggests that CAs might be one of the key links regulating the carbon and nitrogen balance. On the other hand, DEGs enriched in the nitrogen metabolism pathway and classified into the NRT family exhibited similar expression patterns under the N0.5, N1, and N10 treatments compared to the N0 treatment. This was also consistent with the treatments that we set up.

The AP2-EREBP TF family unique to plants is widely involved in the regulation of plant physiological functions and signal transduction pathways such as those of salicylic acid, jasmonic acid, ABA and ethylene. AP2-EREBP TFs show a positive response to nitrogen, especially low-nitrogen stress. This point has been demonstrated in many species and organs, including sugarcane^28^, leaves and roots of watermelon^29^, maize roots^30^, *A. thaliana* roots^31^ and maize^32^. In this study, there was also a positive response to moderate amounts of nitrogen and excessive nitrogen stress in these TFs. This finding supports the fact that the AP2-EREBP TF family plays a functional regulatory role in the plant response to different concentrations of nitrogen. It is worth mentioning that ref0030344-processed-gene-0.2 from the AP2-EREBP TF family was regulated by both nitrogen utilization and excessive nitrogen stress. The function of these TFs in regulating the molecular mechanisms of the plant response to nitrogen deserves to be discussed in the future. The MYB family is one of the largest families of TFs in plants and is widely involved in the regulation of the cell cycle^33^, secondary metabolism^34^, and the responses to biotic and abiotic stress^35^. SiMYB3 in *Setaria italica* plays a key role in regulating root growth, which in turn leads to increased tolerance to low-nitrogen stress^36^. Here, four MYB TFs were shown to play a functional role in nitrogen utilization. This is consistent with our results.

NAC TFs not only participate in plant growth and development (such as lateral root development, flower development, secondary wall formation and plant hormone signal transduction) but also play an important regulatory role in plant responses to abiotic stress, such as drought, salinity, water logging and low temperature^37^. Some members of the NAC TF family are critical in enhancing tolerance to N deficiency in cotton^38^. In this study, one NAC TF (ref0030817) was found to perform a regulatory function in the excessive nitrogen stress response. This may provide a new approach for understanding the role of the NAC TF family in excessive nitrogen stress. A similar phenomenon occurs in the LOB and HB TF families (novel101854, ref0036657). LOB TFs are mainly involved in the regulation of plant morphological development and photosynthesis. For example, MtLOB, induced by nodule inception, regulates lateral root development in *Medicago truncatula*^39^. However, the potential regulatory mechanisms of LOB TFs in the excessive nitrogen stress response are unclear.

The HB TF is a very important regulatory protein in plants that plays an important regulatory role in plant morphogenesis, hormone responses, and biotic and abiotic stress responses. BvHb1.2 has high NOD-like activity in seeds, and BvHb1.1 exhibits a similar ability to use NO for nitrate formation to protect chloroplasts from the deleterious effects of NO^40^. In this study, the HB TF (ref0036657) screened as being related to excessive nitrogen stress may be involved in some similar metabolic mechanisms of nitrite in perennial ryegrass, which could be a key factor for regulation of nitrate accumulation in ryegrass.

The findings of the transcriptome analysis could shed new light on the mechanism underlying the nitrogen utilization efficiency of perennial ryegrass and contribute to breeding projects in the future. For instance, CAB1R (osa:4346803) and CAB2R (osa:4324599) from the photosynthesis-antenna protein pathway and NCED|osa:4333566 and CCD|osa:4330451 from the carotenoid biosynthesis pathway were found to be induced by nitrogen. All these results indicated that the regulatory mechanisms of nitrogen and photosynthesis were closely related in ryegrass. The steroid biosynthesis pathway was the most sensitive to excessive nitrogen stress, rather than the nitrogen metabolism pathway and NRT family, which we originally expected to be the most sensitive. In addition, an AP2-EREBP TF (ref0030344-processed-gene-0.2) with a positive response to both nitrogen absorption and excessive nitrogen stress might play a key role in nitrogen regulation.

In the future, we will further clarify the functions and regulatory mechanisms of these candidate genes in the nitrogen response through reverse genetic methods^40^. Then, according to the regulatory modes of different genes, the corresponding optimal methods, such as overexpression^41^, CRISPR/Cas9^42^ and RNAi^43^, could be used to improve the nitrogen use efficiency of ryegrass. On the other hand, some key genes could also be used to develop molecular markers to evaluate the nitrogen use efficiency of different ryegrass germplasms. Both genetic engineering and molecular marker-assisted selection would make important contributions to promoting the breeding of ryegrass^44,45^.

## Conclusions

At different concentrations of nitrogen, the transcriptional characteristics of a total of 101,376 unigenes were collected in perennial ryegrass by RNA-Seq, among which 345 and 104 DEGs were found to be related to nitrogen utilization and excessive nitrogen stress, respectively.

KEGG enrichment analysis identified four significantly enriched pathways in response to nitrogen at different concentrations, including the photosynthesis-antenna protein, steroid biosynthesis, carotenoid biosynthesis and C5-branched dibasic acid metabolism pathways. A total of 209 members of this transcriptome that were homologous to the NRTs in *Arabidopsis* were extracted, and 104 of them were differentially expressed in each treatment group. In addition, 185 TFs from 37 families, including the top three TF families (AP2-EREBP, MYB, NAC), were identified as being related to nitrogen. Twenty-two TFs from 10 families, especially the AP2-EREBP and MYB families, were considered to perform some important regulatory functions in nitrogen utilization. There were 4 TFs related to excessive nitrogen stress, belonging to the LOB, NAC, AP2-EREBP and HB families. These results provide new insight into the regulatory mechanism of the plant response to nitrogen utilization and excessive nitrogen stress.

## Methods

### Plant materials

The plant material used in this research was perennial ryegrass "Taya" (variety registration no.:285), which was introduced by Professor Liebao Han of Beijing Forestry University from DLF SEEDS A/S Company in Denmark on December 8, 2004. The seeds of perennial ryegrass were preserved at the Lawn Research Institute of Beijing Forestry University (Beijing, China). The collection, preservation and use of plant materials involved in this study complied with relevant institutional, national, and international guidelines and legislation. After a week of vernalization in a 4 °C refrigerator, the seeds were placed on petri dishes with sterile filter paper for germination under natural conditions in the laboratory. After two weeks of seed cultivation, seedlings approximately 5 cm in height were selected and suspended in Hoagland nutrient solution with nitrogen control (Table 1). The seedlings were grown in Hoagland nutrient solution with 0, 0.5, 1, and 10 times the standard concentration of nitrogen in a chamber with 16 h light/8 h darkness at 23 °C for 4 weeks (Table 2); the treatments were named N0, N0.5, N1, and N10, respectively. Three plants from each treatment were randomly selected as biological replicates. A total of 12 samples were immediately treated with liquid nitrogen and stored at − 80 °C.

**Table 1:** Components of the standard Hoagland nutrient solution.

	Molar concentration mmol/L	Mass concentration mg/L	Stock solution concentration g/L (*500)
**A: Major element**			
MgSO_4_·7H_2_O	2.50	616.18	308.09
KH_2_PO_4_	2.00	272.18	136.09
CaCl_2_	5.00	554.90	277.45
KCl	5.00	372.75	186.38
NH_4_NO_3_	7.50	600.00	300.00
**B: Minor element**			
EDTA·FeNa	0.02	8.422	8.422
MnSO_4_·H_2_O	0.006722	1.136	1.136
CuSO_4_·5H_2_O	0.000316	0.079	0.079
ZnSO_4_·7H_2_O	0.000765	0.220	0.220
H_3_BO_3_	0.04625	2.860	2.860
H_2_MoO_4_	0.0005	0.090	0.090

A and B represent the components of the major and minor elements, respectively.

**Table 2:** Nutrient element components in different treatments.

Component mL/L	N0	N0.5	N1.0	N10
MgSO_4_·7H_2_O	2	2	2	2
KH_2_PO_4_	2	2	2	2
CaCl_2_	2	2	2	2
KCl	2	2	2	2
EDTA·FeNa	1	1	1	1
Microelement	1	1	1	1
NH_4_NO_3_	0	1	2	20

*After mixing the ingredients; volume in 1 L of sterile water.

### Phenotypic characterization

Four plants were randomly selected from each treatment as biological replicates, and their height, root length, fresh weight and tiller number were measured. Approximately 0.1 g of each sample was placed in 95% alcohol and then kept in the dark for 24 h, and the specific mass was recorded. The absorbance values of these samples were measured at wavelengths of 665, 649 and 470 nm and were corrected with the value for 95% alcohol. The chlorophyll a/b and total chlorophyll levels were calculated by the corresponding formulas^46^.

### RNA isolation and detection

Total plant RNA was extracted using an RNA extraction kit (OMEGA, Georgia, USA, No. R6827-01) and then treated with the RNase-Free DNaseI Set to remove contaminating genomic DNA. Four methods were used to determine the quality of the RNA as follows. Agarose gel electrophoresis was used to analyse the degradation degree and contamination of the RNA. The purity of the RNA was determined by a Nanodrop (od260/280 ratio). The RNA concentration was accurately quantified by a Qubit. The integrity of RNA was precisely determined by an Agilent 2100.

### Library construction for transcriptome sequencing

After the samples passed the test, the mRNA of the samples was enriched by magnetic beads with oligo-(dT) binding with the polyA tail of mRNA through A-T complementary pairing. Second, the mRNA was fragmented into short segments using fragmentation buffer. Using the mRNA as a template, the first strand of cDNA was synthesized using random hexamers, and then the second strand of cDNA were synthesized using buffer, dNTPs and DNA polymerase I. AMPure XP beads were used to purify the double-stranded cDNA. The purified double-stranded cDNA was subjected to end repair, followed by the addition of an A tail and connection of sequencing joints. AMPure XP beads were used to select each segment size. Finally, PCR enrichment was carried out to obtain the final cDNA library.

After the construction of the library, Qubit2.0 was used for initial quantification, and the library was diluted to 1 ng/µl. Then, the insert size of the library was determined by an Agilent 2100. After the insert size met the expectations, qPCR was used to accurately quantify the effective concentration of the library (> 2 nM) to ensure its quality.

HiSeq sequencing was carried out after pooling different libraries in accordance with the requirements for effective concentration and target removal data.

### Quality control, read mapping and functional annotation

The original image data files obtained by high-throughput sequencingwere transformed into sequenced reads, called raw data, by CASAVA base calling analysis. The error rate of each base sequence was obtained from the Phred value (Phred score, Qphred) using the formula (formula 1: Qphred = − 10log10(e)). The segregation of bases A/T and C/G was confirmed by a base C/G content distribution test. To ensure high quality of the data for analysis, we filtered out low-quality reads with connectors from the raw data and obtained clean reads for further analysis.

HISAT software was selected to localize the filtered sequences with the default parameters^47^. Clean reads were compared to the reference genome (http://185.45.23.197:5080/ryegrassgenome)^48^. The density of total reads mapped to each chromosome in the genome (plus or minus chains) was analysed using StringTie software^49^. Integrative Genomics Viewer was used to visually browse the bam files containing RNA-Seq reads for genomic alignment and the corresponding reference genome and annotation files.

### Analysis of DEGs

DESeq^50^ was adopted to normalize the read count first, the software default parameters were used, and the hypothesis testing probability (p value) was calculated according to the model. Finally, multiple hypothesis testing and correction were performed to obtain the FDR (error detection rate). The screening criteria for DEGs were p value < 0.05 and |log2(FoldChange)| > 1. The overall distribution of the DEGs was inferred from the volcano map. The number of DEGs and the overlap between the comparison groups are shown in Venn diagrams. The FPKM values of the DEGs under different experimental conditions were taken as the expression levels for hierarchical clustering analysis. The log2 ratios of the relative expression levels of the DEGs were clustered by h-cluster, k-means and SOM. Different clustering algorithms divided the DEGs into several clusters, and the genes in the same cluster had similar expression level variation trends under different processing conditions.

### GO and KEGG enrichment analyses of DEGs

GOseq software^51^ was used in the GO enrichment analysis, which was based on Wallenius noncentral geometric distribution. GO terms with corrected P values less than 0.05 were considered to be significantly enriched by the DEGs.

On the other hand, the KEGG database pathway was taken as the unit, and a hypergeometric test was applied to determine the pathways significantly enriched by the DEGs compared with the whole genomic background. We used KOBAS software to test the statistical enrichment of DEGs in KEGG pathways.

### TF analysis

ITAK software was used to predict the TFs in the DEGs. The TF families and rules defined in the database were used to identify TFs by hmmscan^52^.

### Quantitative real-time PCR analysis

Twenty-four unigenes were selected for quantitative real-time PCR analysis to verify the accuracy of the transcriptome data and the actual expression levels of related genes. The previously extracted total RNA was reverse transcribed into cDNA as a qPCR template. All primers designed with Primer Premier 5.0 software are displayed in Table S12. The *EIF4A* gene from ryegrass was used as an internal reference gene to calculate the relative expression level^53^. Quantitative real-time PCR analysis was performed by the Bio–Rad CFX connected server system. The reaction conditions were set as follows: 95 °C for 10 min; 40 cycles of 95 °C for 15 s and 60 °C for 1 min. Each reaction was completed with four technical replicates. The relative expression levels of all genes were determined by the 2^−ΔΔCt^ method^54^.

## Supplementary Information

Supplementary Table S1.

Supplementary Table S2.

Supplementary Table S3.

Supplementary Table S4.

Supplementary Table S5 A-1.

Supplementary Table S5 A-2.

Supplementary Table S5 B-1.

Supplementary Table S5 B-2.

Supplementary Table S5 C-1.

Supplementary Table S5 C-2.

Supplementary Table S5 D-1.

Supplementary Table S5 D-2.

Supplementary Table S5 E-1.

Supplementary Table S5 E-2.

Supplementary Table S6.

Supplementary Table S7.

Supplementary Table S8.

Supplementary Table S9.

Supplementary Table S10.

Supplementary Table S11.

Supplementary Table S12.

## Data Availability

The datasets generated and/or analyzed during the current study will be available in the NCBI database in Aug. 1st, 2021. The datasets used and/or analyzed during the current study are available from the corresponding author on reasonable request.

## Abbreviations

TFs: Transcription factors
KEGG: Kyoto encyclopedia of genes and genomes
GO: Gene ontology
DEGs: Differentially expressed genes
qRT-PCR: Quantitative real-time PCR
