# Supplementary material for: Transcriptome profiling revealed candidate genes, pathways and transcription factors related to nitrogen utilization and excessive nitrogen stress in perennial ryegrass
Source: Sci Rep. 2022 Mar 1;12:3353. doi: 10.1038/s41598-022-07329-7 (PMC8888628; doi:10.1038/s41598-022-07329-7)
Supplement: Supplementary file 4 — Supplementary Table S4. [file 41598_2022_7329_MOESM4_ESM.pdf]

table S4 Information of biosample and bioproject in NCBI database

A

Accession	Sample Name	SPUID	Organism	Tax ID	Cultivar	Isolate	BioProject	Object IDs and corresponding URLs
SAMN15935733	N0-1	N0-1	Lolium perenne	4522	Taya	not collected	PRJNA660099	15935733: https://www.ncbi.nlm.nih.gov/sra/15935733
SAMN15935734	N0-2	N0-2	Lolium perenne	4522	Taya	not collected	PRJNA660099	15935734: https://www.ncbi.nlm.nih.gov/sra/15935734
SAMN15935735	N0-3	N0-3	Lolium perenne	4522	Taya	not collected	PRJNA660099	15935735: https://www.ncbi.nlm.nih.gov/sra/15935735
SAMN15935736	N0.5-1	N0.5-1	Lolium perenne	4522	Taya	not collected	PRJNA660099	15935736: https://www.ncbi.nlm.nih.gov/sra/15935736
SAMN15935737	N0.5-2	N0.5-2	Lolium perenne	4522	Taya	not collected	PRJNA660099	15935737: https://www.ncbi.nlm.nih.gov/sra/15935737
SAMN15935738	N0.5-3	N0.5-3	Lolium perenne	4522	Taya	not collected	PRJNA660099	15935738: https://www.ncbi.nlm.nih.gov/sra/15935738
SAMN15935739	N1-1	N1-1	Lolium perenne	4522	Taya	not collected	PRJNA660099	15935739: https://www.ncbi.nlm.nih.gov/sra/15935739
SAMN15935740	N1-2	N1-2	Lolium perenne	4522	Taya	not collected	PRJNA660099	15935740: https://www.ncbi.nlm.nih.gov/sra/15935740
SAMN15935741	N1-3	N1-3	Lolium perenne	4522	Taya	not collected	PRJNA660099	15935741: https://www.ncbi.nlm.nih.gov/sra/15935741
SAMN15935742	N10-1	N10-1	Lolium perenne	4522	Taya	not collected	PRJNA660099	15935742: https://www.ncbi.nlm.nih.gov/sra/15935742
SAMN15935743	N10-2	N10-2	Lolium perenne	4522	Taya	not collected	PRJNA660099	15935743: https://www.ncbi.nlm.nih.gov/sra/15935743
SAMN15935744	N10-3	N10-3	Lolium perenne	4522	Taya	not collected	PRJNA660099	15935744: https://www.ncbi.nlm.nih.gov/sra/15935744

B

	ID	Release date
Submission	SUB7282502	2021/8/1
BioProject	PRJNA660099	2021/8/1
